# Adults’ reading engagement and wellbeing in Aotearoa New Zealand

**DOI:** 10.1371/journal.pone.0286706

**Published:** 2023-09-28

**Authors:** Stephen Reder

**Affiliations:** Portland State University, Portland, Oregon, United States of America; University of Essex, UNITED KINGDOM

## Abstract

Education and literacy have long been associated with a range of economic and social outcomes in industrialized societies. Recent research based on large-scale national and international surveys has examined effects of education and literacy on individuals’ social and economic outcomes. This paper takes a further step in understanding the importance of literacy for individuals’ economic and social outcomes by disentangling the effects of two different aspects of literacy, *literacy proficiency* as measured by standardized tests and *reading engagement* as measured by self-reports of everyday reading activities. Using recent nationally representative survey data from New Zealand, multivariate regression models estimate the effects of reading engagement on earnings, health, social trust, political efficacy and civic engagement. Reading engagement has statistical and substantial positive effects on each of these outcomes with the effects of literacy proficiency, education and other important variables held constant. Although these results do not imply a causal relationship between reading engagement and the outcomes, they have important implications for policy and practice in adult education as well as for future research about the role of reading engagement in wellbeing more generally.

## Introduction

This paper examines the role of literacy in individual wellbeing using data from New Zealand’s 2014 Programme for the International Assessment of Adult Competencies (PIAAC) survey. Although there are many possible indicators of individual wellbeing, two variables measured in PIAAC–earnings and overall health status–are widely accepted wellbeing indicators and are core elements of the Organisation for Economic Cooperation and Development (OECD) global Better Life Index [1] as well as New Zealand’s Living Standards Framework [2]. This paper will look at these core outcomes as well as several additional social outcomes measured in PIAAC that are often considered as components of individual wellbeing: social trust, political efficacy, and civic engagement.

The central problem addressed by this paper is disentangling the effects of two different aspects of literacy on wellbeing: reading engagement and literacy proficiency. I frame this problem in the context of three lines of previous research: literacy and wellbeing; literacy proficiency and reading engagement; and practice engagement theory.

### Literacy and wellbeing

Education and literacy have long been believed to be central to our social and economic wellbeing [3, 4]. In addition to the education individuals attain in their first cycle of schooling, their continued education as adults is also associated with better life outcomes and wellbeing [5–8]. There is growing evidence from numerous international and national surveys that literacy skills are also associated with a range of economic and social outcomes.

Hanushek and colleagues [9] highlighted the economic returns to cognitive skills such as literacy and numeracy using Mincer-like wage equation models. A variety of social outcomes including health, social trust, political efficacy and civic engagement have been examined in surveys such as the Adult Literacy and Lifeskills (ALL) survey, the International Adult Literacy Survey (IALS) and most recently the PIAAC. Analyses of these survey data generally find that both education and literacy skills are associated with better social outcomes [5, 10–16]. In these studies, individuals with high levels of assessed literacy proficiency are more likely to have positive social outcomes, even after controlling for demographic and educational attainment variables. Dinis da Costa and colleagues [11] analysed the four social outcomes of interest here for countries in the European Union and found literacy proficiency to be more important than education as determinants of these key social outcomes.

Previous research with New Zealand’s PIAAC data has found that adults with higher levels of educational attainment and adults with higher literacy proficiency earn higher wages on average [17]. Scott [18] examined social and wellbeing outcomes in New Zealand and similarly found that adults with more formal education and adults with higher levels of literacy proficiency tend to have higher levels of positive social outcomes. Jones and Satherley [19] and Satherley [20] extended these results to the Māori and Pasifika subpopulations, respectively.

The mechanisms and processes linking literacy proficiency to these social outcomes are complex and may well differ across outcomes. Some possibilities are discussed in [3, 12]. There is widespread consensus among researchers that literacy and other information processing skills are linked to various forms of political participation (e.g., [21]). There is also a substantial research base in health literacy that connects information-processing skills with health, although there is far more research about how skills are used for accessing health information than for communicating with health-care providers or managing one’s own health and care (e.g., [22, 23]).

### Literacy proficiency and reading engagement

Including standardised cognitive assessments in large-scale surveys has enabled analysts to better understand the joint effects of education and cognitive skills on economic and social outcomes. There is also need to consider the *use* of cognitive skills in everyday life and work as a determinant of social and economic outcomes. The impact of an individual’s literacy proficiency, for example, may depend considerably on how much the individual uses reading and writing skills at work and outside of work.

Desjardins and Rubenson [24] analysed ALL data to examine skill use in the workplace. Their analyses along with initial analyses of skill use in the PIAAC data [12] demonstrate how useful such data can be in the context of large-scale assessments. Analyses of the skill use data in both surveys showed substantially increased earnings for workers at higher levels of skill use. With the ALL data, it was estimated that 32%, 20% and 10% increased earnings for high levels of reading, writing and maths skill use at work, respectively, compared to low levels of skill use after controlling for proficiencies, demographics, education, work experience, occupation and industry [24] With the more sophisticated measurement of skill use available in PIAAC, more comprehensive understandings of the relationship between skill use, proficiency and a range of social and economic variables become possible.

Since skill use measures in PIAAC are positively correlated with both education and literacy proficiency [12], multivariate modelling is needed to tease apart the effects of education, proficiency and skill use (practice engagement) on social and economic outcomes. Subsequent research estimated multivariate regression models of various social and economic outcomes as dependent variables, using education, proficiency and skill use and other control variables as independent variables. For most of the Round 1 PIAAC countries, there are significant positive effects of skill use on outcomes when effects of the other variables were statistically controlled [25, 26]. New Zealand’s Round 2 PIAAC data was not yet available for those analyses.

### Practice engagement theory

Practice engagement theory (PET) provides a theoretical framework for these analyses. Practice engagement theorists [27–31] have examined how literacy proficiency, for example, develops during adulthood. PET describes how engagement in reading practices in everyday life (whether at work or outside of work contexts) influences literacy proficiency development over the adult lifespan. PET was initially developed in cross-cultural and cross-situational qualitative research about literacy practices and proficiencies, finding that literacy proficiency and engagement in literacy practices reciprocally influence each other’s development over time [28]. Quantitative modelling of PET became possible as large-scale national and international surveys measured both literacy proficiencies and the use of those skills in everyday activities [31–33]. These initial cross-sectional analyses of relationships between literacy skills and practices were extended by examining age cohorts synthetically aligned across a series of nationally representative surveys. These synthetic cohort studies again found that engagement in literacy practices is associated with population-level growth of literacy proficiency over time [27].

PET has been more rigorously tested in longitudinal panel studies of individual literacy development. The Longitudinal Study of Adult Learning followed a random sample of adults with a low level of education in a metropolitan area in the United States over eight years with repeated measurements of both literacy proficiency and engagement in reading and writing practices [29]. Adult literacy development was also examined in the German National Educational Panel Study [34]. A third panel study in this line of inquiry was PIAAC-L, a longitudinal extension of the PIAAC survey in Germany [30]. Analyses of each panel study found that engagement in reading practices predicted growth of individuals’ literacy proficiency over time, even though the three panel studies involved different measures of literacy proficiency and different measures of engagement in reading practices. Despite these differences in measures, the three studies found positive effects of individuals’ reading engagement on their literacy proficiency growth over time.

This paper further explores PET by examining the relationship of individuals’ reading engagement to their social and economic outcomes. Using cross-sectional modeling of New Zealand’s PIAAC data, I will estimate effects of reading engagement on individual’s earnings, health, social trust, political efficacy and civic engagement, holding literacy proficiency, education, and other variables constant. I hypothesize that reading engagement is positively associated with each of the five outcome variables with the other variables controlled. Although direct causality cannot be inferred through such cross-sectional modelling, empirical tests of these multivariate models can be an important step in increasing our understanding of reading engagement. The empirical estimation of the models illustrates how reading engagement may foster not only the growth of literacy proficiency but also of individuals’ social and economic outcomes. In New Zealand, these questions are important for the adult population in general and especially for the Māori and Pasifika populations who experience disparities in education, literacy and a range of social and economic outcomes [35].

## Methods

The research design involves a secondary analysis of the nationally representative PIAAC survey data collected from New Zealand in 2014. Multivariate regression models estimate, for each of the economic and social wellbeing outcomes, the net predictive effects of reading engagement on the outcome after controlling for effects of literacy proficiency, education and other individual characteristics.

### Data

In the first cycle of PIAAC, 38 countries including New Zealand participated in one of three rounds of data collection between 2012 and 2017. In each country, a household survey was conducted, nationally representative of its adult population aged 16–65. The survey included an extensive background questionnaire (BQ) covering a wide range of topics, including demographics, education, employment, health, and the use of skills at work and in everyday life. The survey also assessed respondents’ literacy, numeracy and problem-solving skills. General descriptions of the survey methods and results are available in (12–14), including how reliability and validity were determined through analysis of extensive field testing results

The data used here are from the New Zealand PIAAC survey, collected in 2014 as part of the second round. Conducted in English, it was administered to a nationally representative sample of 6,177 adults, age 16–65. Individuals in the target age range were randomly selected from households according to a multi-stage national sampling design. The target population from which survey participants were sampled had size 2,749,719. Individuals participated in the survey after giving their informed consent. Further information about New Zealand’s survey is available in [17]. All data were fully anonymised before the author accessed them.

All variables used in analyses except literacy proficiency are based on responses to BQ items. Literacy proficiency, detailed below, was estimated from responses given in a standardized cognitive assessment.

### Outcome variables

Respondents reported gross monthly earnings in 2014 New Zealand dollars. The four social outcome variables were recoded from 5-point Likert response scales into binary outcome variables indicating a high level of general health, social trust, political efficacy and civic engagement. Further details about these outcome variables are presented in the Analytical Methods sections below.

#### Reading engagement

The BQ included self-reported frequencies of performing eight specific reading activities:

How often do you read…

- directions or instructions
- letters, memos or mail
- newspapers or magazines
- professional journals or publications
- books
- manuals or reference materials
- financial statements
- diagrams or schematics

Respondents indicated, on a Likert scale, how often they performed each activity:

- Never
- Less than once a month
- Less than once a week but at least once a month
- At least once a week but not every day
- Every day

All respondents were asked how often they did each reading activity outside of work, and, if they were currently working, how often they did each reading activity at work. I derived two reading engagement indices based on the reported frequencies for work and outside-of-work contexts. For those who were currently employed, I estimated a reading engagement at work (RW) index from the frequencies of reading activities reported for work. The RW index is similar to the READWORK index that OECD derived for its PIAAC data set. I made a separate RW index for two reasons: (1) it is scaled from New Zealand’s data only rather than from the extensive cross-national PIAAC data; (2) the READWORK index was not calculated (and set to missing) for workers who responded “Never” to all eight reading items (i.e., did not read at all in the workplace). The RW index includes those workers in its scaling and so that they can be included in the analyses reported here. Among individuals with both RW and READWORK measures defined, the two are correlated very highly (r = 0.973).

I also estimated a life-wide reading engagement index (RE) that has no counterpart in the OECD skill use measures. I scaled RE from the cross-context activity frequencies reported for the work and outside-of-work contexts. The cross-context task frequency is defined as the greater of the at-work and outside-of-work frequencies reported for the given task. For example, if an individual reported reading newspapers or magazines “every day” at work and “once a week” outside of work, then the cross-context frequency would be “every day”. For individuals who were not employed at the time of the interview, the cross-context frequency was simply the outside-of-work frequency.

Responses were merged across contexts in this way for several reasons. First, the overall cross-context level of practice engagement is of theoretical interest within PET. Second, there are indications that individuals who are working tend to substitute some reading behaviours between non-work and work contexts. Finally, the merged context measures enable analysis of the entire adult population rather than just the currently employed subpopulation. Similar cross-context measures of practice engagement have been used in earlier PIAAC publications (e.g., [30]).

I applied the generalised partial credit model of Item Response Theory [36, 37] to scale the cross-context task frequencies into the RE index and, for respondents who were currently employed, the at-work frequencies into the RW index. The partial credit model of item response theory estimates values of an underlying variable (e.g., overall reading engagement) from ordered responses (e.g., *Never; Less than once a month; Less than once a week but at least once a month; At least once a week but not every day; Every day*) to a set of individual items (e.g., how often individuals perform each of the queried reading tasks). These continuous index variables were scaled to have means of 0 and standard deviations of 1. The scales are based on both the breadth of reading tasks individuals perform and the frequency with which they perform them. The highest quintile of the RE index, for example, is attained only by individuals who perform all eight of the reading tasks at least once a week, whereas the lowest quintile is attained only by individuals who have *ever* performed no more than 2 of the tasks.

#### Literacy proficiency

After completing the BQ, respondents took standardised assessments of literacy, numeracy and problem-solving skills. Of interest here is the literacy assessment, conceptualised within a framework developed by the PIAAC Expert Literacy Group [38]. This framework considers literacy as the ability to understand, evaluate, use and engage with written texts to get everyday things done. The assessment involves only reading, no writing was involved.

The assessment was based on respondents’ answers to sets of cognitive items of varying difficulties. From these responses, ten plausible values were imputed for each respondent’s literacy proficiency, on a 0–500 point scale. By estimating the analytical models with each of these 10 plausible values, measurement error present in the literacy assessment can be accounted for. Further information about the literacy assessment framework, scaling methodology, descriptive anchors and sample items used in PIAAC are available in [12, 38].

### Covariates

- Education (continuous, years)
- Age (continuous, years)—used in social outcomes but not in earnings models
- Work experience (continuous, years)–used in earnings but not in social outcomes models
- Currently employed (binary flag)–used in social outcomes but not in earnings models
- Female (binary)
- New Zealand born (binary)
- Native English speaker (binary)
- Māori identity (binary)
- Pasifika identity (binary)
- NZ European identity (binary)
- Asian identity (binary)

Survey respondents were asked to in an open-ended question to mention all the ethnic groups with which they identified. Their responses were subsequently recoded into the four binary indicators listed above.

### Analytical methods

I estimate a series of multivariate regression models for each outcome variable. I use linear regression models for the continuous outcome (earnings) and logit regressions for the binary social outcomes (high levels of health, social trust, political efficacy and civic engagement). For each outcome, I estimate four models: a *baseline* model that uses a set of covariates as independent variables; a *literacy* model that adds literacy proficiency as an independent variable to the baseline model; a *reading* model that adds reading engagement as an independent variable to the baseline model (RW added to the earnings models, RE to the social outcomes models); and the *full* model that adds both literacy proficiency and reading engagement to the baseline model.

#### Earnings models

The dependent variable is the natural logarithm of self-reported total monthly earnings (gross wages and bonuses). The dependent variable is trimmed at the 1^st^ and 99^th^ percentile to minimise any undue influence of outliers. The earnings models are based on Mincer-style wage equations, with the baseline model including covariates of education, gender, work experience, nativity and ethnicity. Robust linear regression is used to estimate these models for the population of prime age (25–54) workers working full-time (30 or more hours per week). Part-time and self-employed workers are excluded.

#### Social outcome models

For each social outcome, I estimate logit regressions models for the population of adults age 25–65. Dependent variables in these models are derived from responses to a single BQ question having a five-point Likert response scale, recoding binary indicators of a high level of the given outcome. For consistent modeling across outcomes, the highest two scale points of each outcome scale were recoded as 1s and the lower 3 scale points as 0s.

The dependent variable in the health models is derived from the respondent’s self-reported general health status: “Excellent, Very Good, Good, Fair, Poor.” This self-reported health variable has been widely used in other surveys and health research, and has been validated against a range of objective health measures [39–42]. I recoded the five-point Likert response scale into a binary indicator of high health status: “Excellent” or “Very Good” = 1; “Good”, “Fair” or “Poor” = 0.

In the social trust models, the dependent variable is derived from the extent to which the respondent believes “You can trust only a few people”. I recoded the five-point Likert response scale into a binary indicator of high social trust: “Strongly Disagree” or “Disagree” = 1; “Neither Agree nor Disagree”, “Agree” or “Strongly Agree” = 0.

In the political efficacy models, the dependent variable is derived from the response given to “You have no influence on the government”. I recoded the five-point Likert response scale into a binary indicator of high political efficacy: “Strongly Disagree” or “Disagree” = 1; “Neither Agree nor Disagree”, “Agree” or “Strongly Agree” = 0.

In the civic engagement models, the dependent variable is derived from the response to “How often do voluntary work for nonprofit organisations?” I recoded the five-point Likert response scale into a binary indicator of high civic engagement: “Every Day” or “At Least Once a Week but Not Every Day” = 1; “At Least Once a Month but Less Than Once a Week”, “Less than Once a Month” or “Never” = 0).

The continuous independent variables in all earnings and social outcomes models (literacy proficiency, reading engagement, education, age, work experience) are standardised to facilitate comparison of the magnitude of their effects on the dependent variables. All descriptive statistics and model parameters are estimated using Stata 16 and its REPEST procedure [43] that takes into account both the complex sample design of the PIAAC survey and the measurement error in literacy proficiency.

## Results

### Earnings

Monthly earnings are modelled for the subpopulation of fulltime workers (i.e., those working 30 or more hours per week, excluding self-employed) age 25–54 in New Zealand. The subsample of 1,747 represents a subpopulation of size 854,090. The dependent and independent variables are listed in Table 1, showing subpopulation means and standard errors. Workers report a median income of $4,833. About 42% of the workers were female. Workers averaged 14.3 years of education and 19.4 years of work experience. About two in three workers (66%) were born in New Zealand and 4 in 5 (80%) were native English speakers. Slightly more than 1 in 8 workers (12.9%) identified as Māori, 6.5% as Pasifika, 72.6% as NZ European and 15.1% as Asian (Because individuals could identify with multiple ethnicities, these percentages do not add to 100). Workers’ mean literacy proficiency was 289 and their reading engagement (RW) averaged 0.271.

**Table 1:** Descriptive statistics of variables used in models of monthly earnings.

	Type	Mean	Standard Error
*Dependent Variable*			
Monthly Earnings (NZ$)	Continuous	4833*	54.3
*Independent Variables*			
Literacy Proficiency	Continuous	289	1.19
Reading Engagement at Work (RW)	Continuous	0.271	0.0228
Female	Binary	0.421	0.00941
Work Experience (years)	Continuous	19.4	0.207
Education (years)	Continuous	14.3	0.0759
Native English Speaker	Binary	0.800	0.00947
NZ Born	Binary	0.660	0.0122
Māori	Binary	0.129	0.00682
Pasifika	Binary	0.0652	0.00518
NZ European	Binary	0.726	0.0117
Asian	Binary	0.151	0.00804
*N*		1,747	

New Zealand PIAAC 2014, population estimates for fulltime workers, age 25–54, excluding self-employed

Robust standard errors in parentheses

*median rather than mean value shown

Table 2 displays the regression results for log(earnings). Robust regression estimates are shown in the four columns for the baseline, literacy, reading and full models. The independent variables are shown in the first column. Continuous variables–literacy proficiency, reading engagement at work, work experience and education—are standardised to facilitate comparison and interpretation of model coefficients. All other independent variables are binary. The four models have the same independent variables except for the combination of literacy proficiency and reading engagement in each model.

**Table 2:** Linear regression models of (log) earnings.

	Baseline	Literacy	Reading	Full
Literacy Proficiency		0.0959***		0.0809***
		(0.0128)		(0.0118)
Reading Engagement at Work			0.132***	0.122***
			(0.0130)	(0.0129)
Work Experience	0.381***	0.361***	0.325***	0.312***
	(0.0715)	(0.0716)	(0.0694)	(0.0691)
Work Exp. Squared	-0.000476***	-0.000417**	-0.000401**	-0.000357**
	(0.000132)	(0.000130)	(0.000127)	(0.000125)
Female	-0.253***	-0.253***	-0.261***	-0.260***
	(0.0196)	(0.0197)	(0.0177)	(0.0179)
Education	0.197***	0.153***	0.155***	0.122***
	(0.0107)	(0.0119)	(0.0128)	(0.0136)
Native English Speaker	0.0607	0.0438	0.0359	0.0236
	(0.0375)	(0.0385)	(0.0350)	(0.0354)
New Zealand Born	-0.00288	-0.0133	0.00407	-0.00529
	(0.0296)	(0.0293)	(0.0282)	(0.0278)
Māori	-0.0496	-0.0500	-0.0525	-0.0526
	(0.0322)	(0.0317)	(0.0294)	(0.0292)
Pasifika	0.0472	0.0844*	0.0410	0.0729*
	(0.0415)	(0.0392)	(0.0379)	(0.0353)
NZ European	0.0952**	0.0522	0.102***	0.0656*
	(0.0327)	(0.0346)	(0.0300)	(0.0317)
Asian	-0.110*	-0.0886	-0.113*	-0.0943*
	(0.0479)	(0.0466)	(0.0443)	(0.0433)
Constant	8.704***	8.720***	8.650***	8.667***
	(0.0767)	(0.0764)	(0.0683)	(0.0689)
N	1747	1747	1747	1747
r^2^	0.325	0.360	0.388	0.413

Robust standard errors in parentheses

Fulltime workers, age 25–54, excluding self-employed

Literacy Proficiency, Reading Engagement at Work, Work Experience, Education standardised

* *p* < 0.05

** *p* < 0.01

*** *p* < 0.001

As expected from previous research with Mincer-like earnings models, education and work experience have positive effects on wages in the baseline and other models. The significant negative coefficient of work experience squared reflects the gradually declining returns to additional work experience among highly experienced workers. In all models, females receive significantly lower earnings (consistently about 23% lower) than men with other variables controlled. Native speaker and nativity status are not significantly associated with any of the earnings models. In the full model, both Asian and NZ European ethnicities are statistically significant, with Asian having negative and NZ European having positive effects on earnings.

Literacy proficiency and reading engagement at work have statistically significant positive effects on earnings with the effects of education and other covariates controlled. Adding literacy proficiency to the baseline model produces a significantly better fitting model of earnings: F(1, 1746) = 63.88, p = 0.0000. Adding reading engagement to the literacy proficiency-enhanced model again produces a significantly better fitting earnings model: F(1, 1746) = 114.50, p = 0.0000.

The magnitude of the coefficients for literacy proficiency, reading engagement, work experience and education can be compared as effect sizes in the full model since these four variables are standardised. The effect sizes of education, work experience, literacy proficiency and reading engagement are similar, with education being the most potent predictor in the model. Nevertheless, the standardised coefficient for reading engagement of 0.103 corresponds to a substantial 10.3% increase in earnings for each standard deviation increase in reading engagement with other variables controlled.

**Fig 1:**
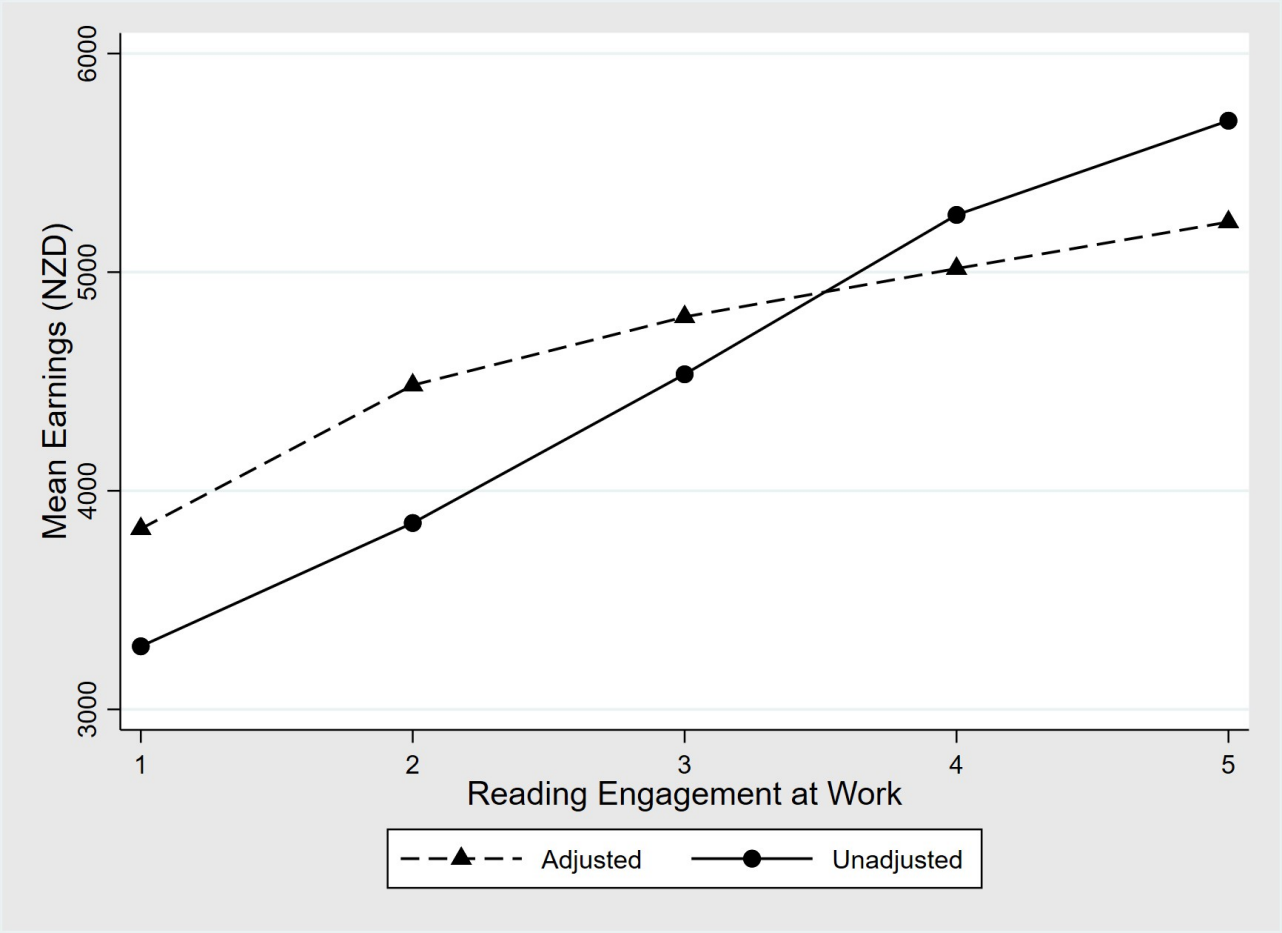
Monthly earnings by reading engagement at work. Mean monthly earnings (NZD) of fulltime workers, age 25–55 (excluding self-employed) are shown as a function of quintile of reading engagement at work. Solid line displays earnings unadjusted by individual characteristics. Dashed line displays earnings adjusted by effects of literacy proficiency, work experience, gender, education, native language, birthplace and ethnicity.

Fig 1 illustrates the effects of reading engagement at work on earnings. Two curves of mean earnings are shown as a function of increasing levels of reading engagement. Reading engagement scores are grouped into five quintiles for this figure. The solid line displays unadjusted mean earnings as a function of reading engagement level. The dashed line shows the model-based estimate of mean earnings after adjusting for effects of literacy proficiency, education work experience and other covariates in the literacy model.

The two curves each show systematic increases in monthly earnings as levels of reading engagement increase. The slope of the adjusted curve is smaller than that of the unadjusted curve, reflecting the positive correlation of reading engagement with other variables positively associated with earnings. Even so, the slope of the adjusted earnings curve is substantial, showing about a 40% marginal increase in earnings across the range of reading engagement levels after correcting for effects of literacy proficiency, education and other variables.

To explore the robustness of the effect of reading engagement at work on earnings, the full model was estimated separately for various occupational groups and firm sizes. Regression results for skilled, semi-skilled white collar, semi-skilled blue collar, and elementary occupational groups are presented in S1 Table. For each occupational group, the reading engagement variable has a statistically significant positive effect on (log) earnings. It is the only statistically significant variable associated with earnings across all occupational groups. S2 Table displays the regression results for five firm sizes: 1–10, 11–50, 51–250, 251–1000 and 1000+ employees. Reading engagement at work has statistically significant positive effects on workers’ (log) earnings in all sizes of firms after controlling for effects of literacy proficiency, work experience, education and other variables. Work experience and education also have statistically significant, positive effects in these models. Literacy proficiency is not statistically significant except in the smaller firm sizes.

### Social outcomes

The binary social outcomes of high levels of health, social trust, political efficacy and civic engagement are modelled for the subpopulation of adults age 25–65. This is a subsample of 4,768 representing a subpopulation of 2,160,818. The dependent and independent variables are listed in Table 3, showing subpopulation means and robust standard errors. Nearly 60% of the adults reported a high health status, more than 25% a high level of social trust, 45% a high level of political efficacy, and 17% a high level of civic engagement (volunteerism). The subpopulation is slightly more than half female, has an average age of 45 years and completed an average of 14 years of schooling. Approximately 70% were born in New Zealand and about 83% are native English speakers. About 81% were employed at the time of their interview. About 1 in 8 (12.9%) reported a Māori identity, 5.6% a Pasifika identity, 75.2% a NZ European identity and 11.9% an Asian identity. The subpopulation’s mean literacy proficiency score was 282 and its mean life-wide reading engagement (RE) index was 0.078.

**Table 3:** Basic statistics of variables in models of social outcomes.

	Type	Mean	Standard Error
*Dependent Variables*			
High Health	Binary	0.598	0.00814
High Social Trust	Binary	0.257	0.00693
High Political Efficacy	Binary	0.452	0.00743
High Civic Engagement	Binary	0.171	0.00667
*Independent Variables*			
Literacy Proficiency	Continuous	282	0.939
Reading Engagement (RE)	Continuous	0.0784	0.0131
Female	Binary	0.524	0.00187
Age	Continuous	44.7	0.0694
Education	Continuous	14.0	0.0508
Native English Speaker	Binary	0.826	0.00472
NZ Born	Binary	0.705	0.00638
Employed	Binary	0.809	0.00663
Māori	Binary	0.129	0.00122
Pasifika	Binary	0.056	0.00217
NZ European	Binary	0.752	0.00434
Asian	Binary	0.119	0.00290
*N*		4,768	

New Zealand PIAAC 2014, population estimates for individuals age 25–65

Table 4 displays results for the logit regression models of high levels of the social outcomes. The table displays the full models for each of the four social outcomes. Complete results—the baseline, literacy, reading and full models—are shown in S3–S6 Tables for health, social trust, political efficacy and civic engagement, respectively. The independent variables used in these models are shown in the first column. Continuous variables–literacy proficiency, life-wide reading engagement, age and education—are standardised to facilitate comparison and interpretation of model coefficients. All other independent variables are binary.

**Table 4:** Logit regression models for full models of social outcomes: High levels of health, social trust, political efficacy and civic engagement.

	Health	Trust	Political	Civic
Literacy Proficiency	0.170***	0.313***	0.313***	0.169*
	(0.0458)	(0.0570)	(0.0570)	(0.0670)
Life-Wide Reading Engagement	0.102**	0.104*	0.104*	0.154**
	(0.0391)	(0.0456)	(0.0456)	(0.0499)
Age	-0.0458	0.0998*	0.0998*	0.323***
	(0.0336)	(0.0442)	(0.0442)	(0.0503)
Age-squared	-0.0209	-0.0732	-0.0732	-0.0677
	(0.0388)	(0.0464)	(0.0464)	(0.0438)
Female	0.197**	0.210*	0.210*	0.312***
	(0.0746)	(0.0874)	(0.0874)	(0.0783)
Education	0.172***	0.181***	0.181***	0.181**
	(0.0461)	(0.0469)	(0.0469)	(0.0576)
Native English Speaker	-0.000979	-0.0625	-0.0625	-0.112
	(0.142)	(0.162)	(0.162)	(0.153)
NZ Born	0.0479	0.159	0.159	-0.0695
	(0.108)	(0.0997)	(0.0997)	(0.131)
Employed	0.602***	0.277**	0.277**	-0.359**
	(0.0800)	(0.0969)	(0.0969)	(0.113)
Māori	-0.490***	-0.142	-0.142	0.253
	(0.116)	(0.145)	(0.145)	(0.164)
Pasifika	-0.249	-0.159	-0.159	0.470*
	(0.153)	(0.232)	(0.232)	(0.229)
NZ European	0.0933	0.303	0.303	-0.538**
	(0.108)	(0.168)	(0.168)	(0.186)
Asian	0.0569	-0.0554	-0.0554	-0.661***
	(0.174)	(0.188)	(0.188)	(0.194)
Constant	-0.189	-1.660***	-1.660***	-0.907***
	(0.164)	(0.176)	(0.176)	(0.234)
*N*	4768	4768	4768	4768

Robust standard errors in parentheses

Individuals age 25–65

Literacy Proficiency, Life-Wide Reading Engagement, Age, Education standardised

* *p* < 0.05

** *p* < 0.01

*** *p* < 0.001

Literacy proficiency and life-wide reading engagement have statistically significant positive effects on earnings with the effects of education and other covariates controlled. Adding literacy proficiency to the baseline model produces a significantly better fitting model for health (F(1, 4767) = 23.98, p = 0.0000), for social trust (F(1,4767) = 41.65, p = 0.0000), for political efficacy (F(1,4767) = 77.52, p = 0.0000), and for civic engagement (F(1,4767) = 11.95, p = 0.0006). Adding reading engagement to the literacy proficiency-enhanced models again produces significantly better fitting models for health (F(1, 4767) = 6.48, p = 0.0109), for social trust (F(1,4767) = 5.35, p = 0.0207), for political efficacy (F(1, 4767) = 22.49, p = 0.0000), and for civic engagement (F(1,4767) = 9.25, p = 0.0024).

There is a consistent pattern of effects across the four outcomes. Both literacy proficiency and life-wide reading engagement have statistically significant positive effects on each social outcome with the effects of education and other covariates statistically controlled. Years of education has a statistically significant positive effect on each outcome while native language and nativity status are not significant predictors of any of the social outcomes. The effects of the other covariates are not consistent across the four social outcomes. Gender and age each have significant effects for 3 of the 4 social outcomes. Age has statistically significant, positive effects on social trust, political efficacy and civic engagement but no significant effect on health status. Being female has statistically significant, positive effects on health, social trust and civic engagement outcomes but no significant effect on political efficacy. Being currently employed has statistically significant, positive effects on health and social trust, a significant negative effect on civic engagement and no significant effect on political efficacy.

The effects of ethnicity on the social outcomes exhibit a mixed picture. With other variables controlled, Māori identity has no significant effect on any of the social outcomes except health status, for which it has a statistically significant negative effect. Pasifika identity has statistically significant positive effects on political efficacy and civic engagement, but no significant effect on health status or social trust. New Zealand Euro identity has a significant positive effect on social trust and negative effect on civic engagement, whereas Asian identity has a significant negative effect on civic engagement.

**Fig 2:**
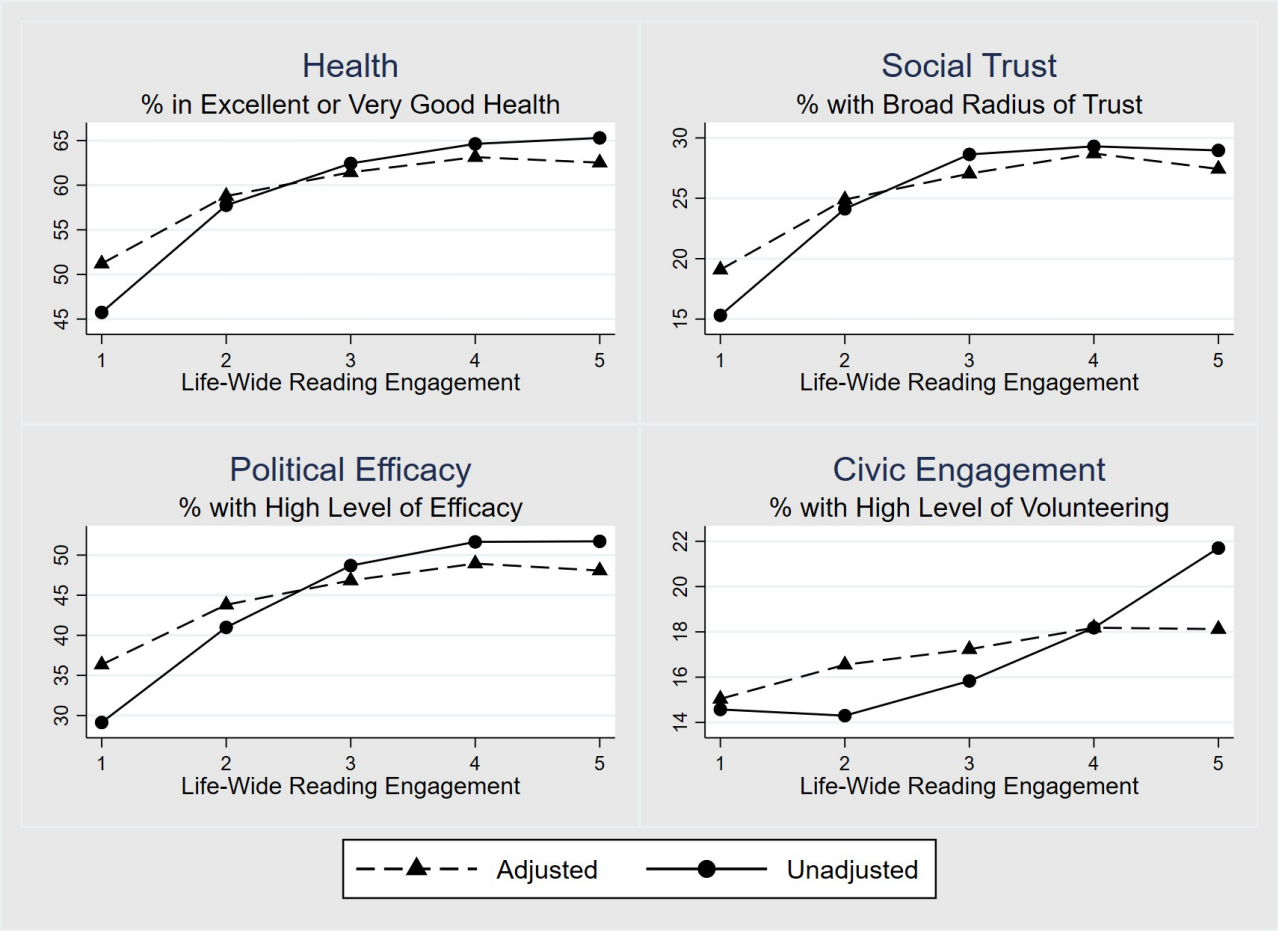
Percentage of individuals, age 25–65, reporting high levels of social outcomes as a function of quintile of life-wide reading engagement. Top left: General health status. Top right: Social trust. Bottom left: Political efficacy. Bottom right: Civic engagement. Solid lines display percentages unadjusted by individual characteristics. Dashed lines display percentages adjusted by effects of literacy proficiency, age, gender, education, native language, birthplace, employment status and ethnicity.

The key finding here is that life-wide reading engagement has a statistically significant positive effect on all four social outcomes with the effects of literacy proficiency and other covariates statistically controlled. Fig 2 illustrates the magnitude of these reading engagement effects on the various social outcomes. Each panel of the figure displays the effects of reading engagement on a particular social outcome: health is shown in the top left panel, social trust in the top right, political efficacy in the lower left, and civic engagement in the lower right panel. In each panel, two curves show the percentage of individuals reporting a high level of the outcome as a function of increasing levels of life-wide reading engagement, with reading engagement levels grouped into five quintiles. The solid lines display the (unadjusted) percentage of individuals reporting a high level of the social outcome across the five levels of reading engagement. The dashed lines show the percentages after adjusting for effects of literacy, proficiency, education and other covariates.

Both unadjusted and adjusted curves show systematic increases in the outcomes as levels of reading engagement increase. Increases in the adjusted curves–whose statistical significance is confirmed by the regression results in Table 4 —are relatively small: the high outcome levels for health increase from 51% to 62%, for social trust from 20% to 27%, for political efficacy from 36% to 45%, and for civic engagement from 15% to 18%. Fig 2 shows that for each social outcome, almost all of the increase attributable to reading engagement occurs from the 1^st^ to 3^rd^ quintile of reading engagement. The social outcome curves appear relatively flat at still higher levels of reading engagement. This flattening is not seen in the earnings curves shown in Fig 1. The flattening of the adjusted social outcomes curves may be of considerable practical importance, as it suggests that interventions designed to increase reading engagement may have beneficial effects on social outcomes for interventions aiming to moderately increase lower levels of reading engagement.

## Discussion

The effects of reading engagement on selected economic and social outcomes in New Zealand were estimated in multivariate regression environments. Monthly earnings of fulltime prime-age workers, age 25–54, were modelled with an index of reading engagement in work settings. Four social outcomes—general health status, social trust, political efficacy and civic engagement—were modelled for adults age 25–65, using a life-wide reading engagement index constructed across both work and non-work settings. The regression results are consistent with a broadened PET framework in which reading engagement is associated with not only the growth of adults’ literacy proficiencies seen in previous research [27, 30] but also with better economic and social outcomes with controls for the effects of literacy proficiency, education and other covariates. The central finding of this paper is that two aspects of literacy contribute to adults’ economic and social outcomes in New Zealand, literacy proficiency and reading engagement.

Reading engagement at work has a statistically significant and substantial positive effect on monthly earnings among fulltime workers with the effects of literacy proficiency, education, work experience and other variables controlled. This is a robust finding for fulltime workers as a whole as well as for those working in numerous occupational categories and firm sizes examined. In the full Mincer-like log earnings model, the standardised coefficient of reading engagement is 0.103, corresponding to a 10.3% increase in earnings for each standard deviation increase of reading engagement. Across the range of reading engagement levels, regression-adjusted mean earnings increase from about $3800 per month among adults in the lowest quintile of reading engagement to $5200 per month in the highest quintile.

Life-wide reading engagement has a statistically significant positive effect on health status after controlling for the effects of literacy proficiency, education and other variables. Previous research (e.g., [44–46]) has made it clear that literacy is a social determinant of health. The findings reported here help extend previous research to New Zealand and elaborate on how individuals’ engagement in reading practices may underlie observed relationships between literacy and health. Life-wide reading engagement also has statistically significant positive effects on the other social outcomes examined after controlling for the effects of literacy proficiency, education, and other variables. This holds for the outcomes of social trust, political efficacy and civic engagement. After adjusting for the effects of the other variables in the models, the percentage of adults reporting a high level of each social outcome increases with the level of their life-wide reading engagement.

Although the increases across levels of reading engagement are statistically significant for both the earnings and social outcomes, they are relatively modest for the social outcomes. The regression-adjusted percentage of adults reporting a high health status increases from 51% among adults in the lowest quintile of life-wide reading engagement up to 62% among those in the highest quintile; the corresponding increases for social trust are from 20% to 27%; for political efficacy from 36% to 45%; and for civic engagement from 15% to 18%. For each of these social outcomes, the increases seen across levels of life-wide reading engagement are evident across only the lower quintiles of the reading engagement scale, in contrast with the substantial earnings gains evident across the entire scale of reading engagement at work.

### Limitations

The significant effects of reading engagement found in the cross-sectional models of earnings and social outcomes do not, of course, imply causal relationships between reading engagement and those outcomes. Additional research is needed to identify causal mechanisms that may underlie the relationships observed here, including instrumental variable models of cross-sectional data and panel models of longitudinally measured outcomes. As noted before, previous research has found literacy to have a causal relationship with both earnings and health status, suggesting that both literacy proficiency and reading engagement may have such causal relationships as well. Previous research has not yet identified causal relationships between literacy and the other social outcomes examined in this paper.

A second important limitation is encountered in trying to interpret the robust and substantial effects of reading engagement at work on monthly earnings. As measured in PIAAC, reading engagement at work is an attribute of the job as well as of the individual who performs the job. In contrast with literacy proficiency, which is conceptualized as an attribute of the individual that moves from job to job (context to context), reading engagement at work is shaped by the design, requirements and affordances of the job and workplace. Research is needed that compares the earnings of workers holding a given job who use different levels of reading engagement in performing their work. Multi-level surveys—in which both firms and jobs are sampled and employees are sampled within those firms and jobs–would be a promising source of needed data.

### Implications

Subject to these limitations, the findings in this paper have important implications for programmes, policy and future research. The findings, considered in the context of previous research, suggest that effective adult literacy programmes may not only improve adults’ literacy abilities, they may help improve key economic and social dimensions of their lives as well. Strong quasi-experimental comparisons of high school dropouts in the United States who participate or do not participate in adult literacy programmes found that participation leads to substantial gains in long-term earnings and other outcomes [47]. The Canadian UPSKILL project, in a random control trial, found substantial impacts of basic skills instruction for incumbent hospitality workers on several outcomes: proficiency, skill use on the job, job performance and employer profits [48].

For many adults, reading engagement is more malleable across the lifespan than either literacy proficiency or educational attainment [29, 31, 49]. Interventions designed to increase reading engagement should be systematically explored and evaluated to see how they affect adults’ wellbeing over time. These interventions could be instructional or non-instructional. Non-instructional interventions might include re-designing workplaces and jobs to foster increased reading engagement at work [50, 51] and increased earnings. Health literacy interventions (e.g., for managing a chronic health condition) that develop medical practices and reading materials that are more engaging for adults, provide a good example of effective non-instructional interventions.

Instructional interventions might be designed to increase reading engagement directly rather than literacy proficiency. Such practice-centred instruction has long been considered a promising approach to adult literacy education in numerous countries [49]. The present findings suggest it may also be an effective means to improve adults’ earnings, health and other dimensions of wellbeing. Contextualising adult literacy instruction with personally meaningful health information and materials, for example, may strengthen reading engagement in ways that improve both health outcomes and literacy over time.

The findings of this study also suggest that initiatives and programmes that connect reading engagement with political efficacy and civic participation may increase both reading engagement and these social outcomes. Contextualising reading engagement in programmes fostering political efficacy and civic engagement–a cornerstone of Freire-inspired pedagogies—has a long tradition in adult education around the world. Fig 2, that graphically illustrates the marginal returns to increased reading engagement on social outcomes, is of interest in this regard. Increased social outcomes are apparent only across the lower portion of the reading engagement scale. This suggests that interventions targeting minimally engaged readers may foster a wide range of improvements in wellbeing. This approach may be particularly effective with adults having literacy challenges, conceptualized here in terms of their level of reading engagement rather than their literacy proficiency (i.e., test scores).

The findings also have implications for education, training and wellbeing policy in New Zealand. The goals and designs of adult literacy programmes and interventions should be formulated in terms of both literacy proficiency and reading engagement. Given the increasing evidence for PET, programme evaluations should measure shorter-term impacts on reading engagement and longer-term impacts on literacy proficiency. This may help policymakers recognize the importance of complex skill formation processes underlying wellbeing frameworks and funding priorities, as called for by Cochrane and colleagues [52]. As evidence continues to grow about its broad impact on social and economic outcomes, reading engagement could be usefully incorporated into wellbeing frameworks in New Zealand and other countries.

Further research is needed to better understand and address the ubiquitous effects of gender observed in these economic and social outcomes. As widely found in previous research, fulltime female workers experience substantial earnings penalties in the current study even with controls for work experience, education, literacy and other variables. At the same time, women appear to attain better social outcomes than men with these variables controlled.

In addition to exploring interventions that foster reading engagement, several lines of additional research will enhance our understanding of how practice engagement is related to social and economic outcomes. Linkages available between New Zealand’s PIAAC data and its national administrative database, the Integrated Data Infrastructure, may provide an opportunity to identify causal relationships between reading engagement and longitudinal earnings and health data [53]. Intervention and experimental research will also help clarify the nature of the mechanisms underlying observed relationships between reading engagement and various outcome measures.

In addition to reading engagement, the PIAAC data include information about adults’ engagement in writing, maths and ICT practices. Engagement in these additional types of practices may also have important effects on social and economic outcomes. Examining the effects of these practice engagement measures on wellbeing outcomes will be useful, both in cross-sectional analyses and in potential longitudinal studies within the Integrated Data Infrastructure.

## Conclusion

This study contributes to the literature on the relationship between cognitive skills and wellbeing, focusing on the relationship between literacy and adults’ economic and social outcomes as measured in New Zealand’s PIAAC survey. The study adds to the literature by showing that two distinct aspects of literacy–literacy proficiency and reading engagement–are positively associated with monthly earnings, general health status, social trust, political efficacy and civic engagement. These associations remain statistically significant after controlling for other variables including education, work experience, age, gender, ethnicity, native language and birthplace.

These observed associations do not imply causal relationships between literacy and the outcomes. When considered in relation to other research, the findings suggest that both literacy proficiency and reading engagement are likely social determinants of earnings, general health status and possibly the other social outcomes examined here. Further research is needed to confirm the causality and mechanisms underlying the observed relationships between reading engagement and these economic and social dimensions of wellbeing.

## Supporting information

S1 Table Linear regression models of log earnings for ISCO occupational groups. (DOCX)

S2 Table Linear regression models of log earnings for five firm sizes. (DOCX)

S3 Table Logit models of high health status. (DOCX)

S4 Table Logit models of high social trust. (DOCX)

S5 Table Logit models of high political efficacy. (DOCX)

S6 Table Logit models of high civic engagement. (DOCX)
