# Supplementary material for: Adults’ reading engagement and wellbeing in Aotearoa New Zealand
Source: PLoS One. 2023 Sep 28;18(9):e0286706. doi: 10.1371/journal.pone.0286706 (PMC10538774; doi:10.1371/journal.pone.0286706)
Supplement: S1 Table — (DOCX) [file pone.0286706.s001.docx]

**S1 Table. Linear regression models of log earnings for ISCO occupational groups**.

|  | Skilled | Semi-Skilled White Collar | Semi-Skilled Blue Collar & Elementary |
| --- | --- | --- | --- |
| Literacy Proficiency | 0.0798^***^ | 0.0760^**^ | 0.0323 |
|  | (0.0173) | (0.0290) | (0.0217) |
|  |  |  |  |
| Reading Engagement at Work | 0.0807^***^ | 0.0974^***^ | 0.0689^***^ |
|  | (0.0183) | (0.0234) | (0.0171) |
|  |  |  |  |
| Work Experience | 0.0995^***^ | 0.0932^***^ | 0.0274 |
|  | (0.0117) | (0.0200) | (0.0308) |
|  |  |  |  |
| Work Exp. Squared | -0.0361^***^ | -0.0146 | -0.00612 |
|  | (0.0109) | (0.0185) | (0.0280) |
|  |  |  |  |
| Female | -0.240^***^ | -0.189^***^ | -0.277^***^ |
|  | (0.0240) | (0.0412) | (0.0449) |
|  |  |  |  |
| Education | 0.108^***^ | 0.0440 | 0.0244 |
|  | (0.0178) | (0.0347) | (0.0238) |
|  |  |  |  |
| Native English Speaker | 0.0247 | 0.0233 | -0.0437 |
|  | (0.0451) | (0.0872) | (0.0739) |
|  |  |  |  |
| New Zealand Born | -0.0120 | -0.0239 | 0.0333 |
|  | (0.0337) | (0.0764) | (0.0470) |
|  |  |  |  |
| Māori | -0.0946^*^ | -0.0108 | -0.0266 |
|  | (0.0395) | (0.0657) | (0.0638) |
|  |  |  |  |
| Pasifika | 0.0907 | -0.00464 | -0.0379 |
|  | (0.0520) | (0.104) | (0.0704) |
|  |  |  |  |
| NZ European | 0.140^**^ | -0.146 | 0.0239 |
|  | (0.0467) | (0.0980) | (0.0600) |
|  |  |  |  |
| Asian | -0.0407 | -0.201 | -0.117 |
|  | (0.0572) | (0.131) | (0.103) |
|  |  |  |  |
| Constant | 8.610^***^ | 8.481^***^ | 8.473^***^ |
|  | (0.0621) | (0.149) | (0.0744) |
|  |  |  |  |
| N | 1012 | 351 | 376 |
| r^2^ | 0.334 | 0.302 | 0.225 |

Standard errors in parentheses

Fulltime workers, age 25-54, not self-employed

Literacy Proficiency, Reading Engagement, Work Experience, Education standardised

^*^ *p* < 0.05, ^**^ *p* < 0.01, ^***^ *p* < 0.001
