# Supplementary material for: Adults’ reading engagement and wellbeing in Aotearoa New Zealand
Source: PLoS One. 2023 Sep 28;18(9):e0286706. doi: 10.1371/journal.pone.0286706 (PMC10538774; doi:10.1371/journal.pone.0286706)
Supplement: S2 Table — (DOCX) [file pone.0286706.s002.docx]

**S2 Table.** **Linear regression models of log earnings for five firm sizes**

|  | 1-10  Employees | 11-50 Employees | 51-250 Employees | 251-1000 Employees | >1000 Employees |
| --- | --- | --- | --- | --- | --- |
| Literacy Proficiency | 0.0853^***^ | 0.103^***^ | 0.0272 | 0.0763 | 0.0298 |
|  | (0.0220) | (0.0216) | (0.0221) | (0.0500) | (0.0670) |
|  |  |  |  |  |  |
| Reading Engagement at Work | 0.0920^***^ | 0.118^***^ | 0.0734^***^ | 0.108^**^ | 0.167^**^ |
|  | (0.0172) | (0.0189) | (0.0177) | (0.0335) | (0.0599) |
|  |  |  |  |  |  |
| Work Experience | 0.0700^***^ | 0.0700^**^ | 0.128^***^ | 0.0772^**^ | 0.103^*^ |
|  | (0.0207) | (0.0232) | (0.0160) | (0.0277) | (0.0417) |
|  |  |  |  |  |  |
| Work Exp. Squared | -0.0352 | -0.00209 | -0.0356^*^ | -0.0961^**^ | -0.0187 |
|  | (0.0192) | (0.0203) | (0.0147) | (0.0325) | (0.0397) |
|  |  |  |  |  |  |
| Female | -0.298^***^ | -0.282^***^ | -0.289^***^ | -0.133^*^ | -0.164 |
|  | (0.0348) | (0.0356) | (0.0319) | (0.0606) | (0.0940) |
|  |  |  |  |  |  |
| Education | 0.0892^***^ | 0.0749^***^ | 0.180^***^ | 0.0786^*^ | 0.169^*^ |
|  | (0.0271) | (0.0201) | (0.0264) | (0.0356) | (0.0697) |
|  |  |  |  |  |  |
| Native English Speaker | 0.0471 | 0.0538 | 0.0240 | 0.0123 | -0.0288 |
|  | (0.0566) | (0.0583) | (0.0636) | (0.119) | (0.126) |
|  |  |  |  |  |  |
| New Zealand Born | 0.00601 | -0.0345 | 0.0414 | -0.0340 | -0.0596 |
|  | (0.0433) | (0.0545) | (0.0522) | (0.0940) | (0.120) |
|  |  |  |  |  |  |
| Maori | -0.144^**^ | -0.0429 | -0.0522 | -0.0160 | -0.0251 |
|  | (0.0556) | (0.0606) | (0.0617) | (0.0867) | (0.127) |
|  |  |  |  |  |  |
| Pasifika | -0.0245 | 0.108 | 0.0820 | -0.0226 | 0.103 |
|  | (0.0884) | (0.0688) | (0.0566) | (0.111) | (0.162) |
|  |  |  |  |  |  |
| NZ European | -0.0550 | 0.0250 | 0.0875 | 0.259^*^ | 0.201 |
|  | (0.0791) | (0.0639) | (0.0667) | (0.103) | (0.165) |
|  |  |  |  |  |  |
| Asian | -0.208^*^ | -0.0799 | -0.0342 | -0.0158 | -0.132 |
|  | (0.0991) | (0.109) | (0.0650) | (0.125) | (0.159) |
|  |  |  |  |  |  |
| Constant | 8.590^***^ | 8.575^***^ | 8.572^***^ | 8.608^***^ | 8.634^***^ |
|  | (0.0863) | (0.0843) | (0.0711) | (0.128) | (0.185) |
|  |  |  |  |  |  |
| N | 450 | 537 | 431 | 200 | 126 |
| r^2^ | 0.395 | 0.380 | 0.478 | 0.358 | 0.349 |

Standard errors in parentheses

Fulltime workers, age 25-54, not self-employed

Literacy Proficiency, Reading Engagement, Work Experience, Education standardised

^*^ *p* < 0.05, ^**^ *p* < 0.01, ^***^ *p* < 0.001
