# Supplementary material for: Adults’ reading engagement and wellbeing in Aotearoa New Zealand
Source: PLoS One. 2023 Sep 28;18(9):e0286706. doi: 10.1371/journal.pone.0286706 (PMC10538774; doi:10.1371/journal.pone.0286706)
Supplement: S3 Table — (DOCX) [file pone.0286706.s003.docx]

**S3 Table. Logit models of high health status.**

|  | Baseline | Literacy | Reading | Full |
| --- | --- | --- | --- | --- |
| Literacy Proficiency |  | 0.188^***^ |  | 0.170^***^ |
|  |  | (0.0436) |  | (0.0458) |
|  |  |  |  |  |
| Life-Wide Reading Engagement |  |  | 0.126^***^ | 0.102^**^ |
|  |  |  | (0.0371) | (0.0391) |
|  |  |  |  |  |
| Age | -0.0688^*^ | -0.0413 | -0.0711^*^ | -0.0458 |
|  | (0.0335) | (0.0333) | (0.0337) | (0.0336) |
|  |  |  |  |  |
| Age-squared | -0.0350 | -0.0224 | -0.0316 | -0.0209 |
|  | (0.0385) | (0.0388) | (0.0385) | (0.0388) |
|  |  |  |  |  |
| Female | 0.188^*^ | 0.190^*^ | 0.196^**^ | 0.197^**^ |
|  | (0.0751) | (0.0744) | (0.0752) | (0.0746) |
|  |  |  |  |  |
| Education | 0.278^***^ | 0.198^***^ | 0.236^***^ | 0.172^***^ |
|  | (0.0419) | (0.0453) | (0.0442) | (0.0461) |
|  |  |  |  |  |
| Native English Speaker | 0.0507 | 0.00756 | 0.0359 | -0.000979 |
|  | (0.141) | (0.144) | (0.139) | (0.142) |
|  |  |  |  |  |
| NZ Born | 0.0578 | 0.0439 | 0.0608 | 0.0479 |
|  | (0.107) | (0.108) | (0.106) | (0.108) |
|  |  |  |  |  |
| Employed | 0.677^***^ | 0.644^***^ | 0.621^***^ | 0.602^***^ |
|  | (0.0793) | (0.0796) | (0.0802) | (0.0800) |
|  |  |  |  |  |
| Maori | -0.505^***^ | -0.490^***^ | -0.504^***^ | -0.490^***^ |
|  | (0.117) | (0.116) | (0.117) | (0.116) |
|  |  |  |  |  |
| Pasifika | -0.299^*^ | -0.233 | -0.312^*^ | -0.249 |
|  | (0.151) | (0.152) | (0.152) | (0.153) |
|  |  |  |  |  |
| NZ European | 0.151 | 0.0844 | 0.154 | 0.0933 |
|  | (0.106) | (0.108) | (0.106) | (0.108) |
|  |  |  |  |  |
| Asian | 0.0150 | 0.0614 | 0.0150 | 0.0569 |
|  | (0.172) | (0.175) | (0.171) | (0.174) |
|  |  |  |  |  |
| Constant | -0.315 | -0.216 | -0.269 | -0.189 |
|  | (0.167) | (0.165) | (0.165) | (0.164) |
|  |  |  |  |  |
| N | 4768 | 4768 | 4768 | 4768 |

Standard errors in parentheses

Individuals age 25-65

Literacy Proficiency, Life-Wide Reading Engagement, Age, Education standardised

^*^ *p* < 0.05, ^**^ *p* < 0.01, ^***^ *p* < 0.001
