# Supplementary material for: Adults’ reading engagement and wellbeing in Aotearoa New Zealand
Source: PLoS One. 2023 Sep 28;18(9):e0286706. doi: 10.1371/journal.pone.0286706 (PMC10538774; doi:10.1371/journal.pone.0286706)
Supplement: S4 Table — (DOCX) [file pone.0286706.s004.docx]

**S4 Table. Logit models of high social trust.**

|  | Baseline | Literacy | Reading | Full |
| --- | --- | --- | --- | --- |
| Literacy Proficiency |  | 0.325^***^ |  | 0.313^***^ |
|  |  | (0.0556) |  | (0.0570) |
|  |  |  |  |  |
| Life-Wide Reading Engagement |  |  | 0.135^**^ | 0.104^*^ |
|  |  |  | (0.0424) | (0.0456) |
|  |  |  |  |  |
| Age | 0.0541 | 0.105^*^ | 0.0501 | 0.0998^*^ |
|  | (0.0443) | (0.0448) | (0.0436) | (0.0442) |
|  |  |  |  |  |
| Age-squared | -0.0964^*^ | -0.0735 | -0.0948^*^ | -0.0732 |
|  | (0.0463) | (0.0466) | (0.0460) | (0.0464) |
|  |  |  |  |  |
| Female | 0.193^*^ | 0.202^*^ | 0.203^*^ | 0.210^*^ |
|  | (0.0872) | (0.0874) | (0.0874) | (0.0874) |
|  |  |  |  |  |
| Education | 0.336^***^ | 0.205^***^ | 0.296^***^ | 0.181^***^ |
|  | (0.0395) | (0.0464) | (0.0414) | (0.0469) |
|  |  |  |  |  |
| Native English Speaker | 0.0121 | -0.0573 | 0.000566 | -0.0625 |
|  | (0.157) | (0.163) | (0.157) | (0.162) |
|  |  |  |  |  |
| NZ Born | 0.179 | 0.155 | 0.183 | 0.159 |
|  | (0.0999) | (0.100) | (0.0993) | (0.0997) |
|  |  |  |  |  |
| Employed | 0.373^***^ | 0.317^***^ | 0.315^**^ | 0.277^**^ |
|  | (0.0933) | (0.0931) | (0.0978) | (0.0969) |
|  |  |  |  |  |
| Maori | -0.181 | -0.144 | -0.178 | -0.142 |
|  | (0.145) | (0.145) | (0.145) | (0.145) |
|  |  |  |  |  |
| Pasifika | -0.249 | -0.142 | -0.267 | -0.159 |
|  | (0.236) | (0.230) | (0.238) | (0.232) |
|  |  |  |  |  |
| NZ European | 0.401^*^ | 0.291 | 0.411^*^ | 0.303 |
|  | (0.169) | (0.168) | (0.169) | (0.168) |
|  |  |  |  |  |
| Asian | -0.132 | -0.0553 | -0.130 | -0.0554 |
|  | (0.192) | (0.189) | (0.192) | (0.188) |
|  |  |  |  |  |
| Constant | -1.812^***^ | -1.677^***^ | -1.779^***^ | -1.660^***^ |
|  | (0.175) | (0.176) | (0.176) | (0.176) |
|  |  |  |  |  |
| N | 4768 | 4768 | 4768 | 4768 |

Standard errors in parentheses

Individuals age 25-65

Literacy Proficiency, Life-Wide Reading Engagement, Age, Education standardised

^*^ *p* < 0.05, ^**^ *p* < 0.01, ^***^ *p* < 0.001
