# Supplementary material for: Adults’ reading engagement and wellbeing in Aotearoa New Zealand
Source: PLoS One. 2023 Sep 28;18(9):e0286706. doi: 10.1371/journal.pone.0286706 (PMC10538774; doi:10.1371/journal.pone.0286706)
Supplement: S5 Table — (DOCX) [file pone.0286706.s005.docx]

**S5 Table. Logit models of high political efficacy.**

|  | Baseline | Literacy | Reading | Full |
| --- | --- | --- | --- | --- |
| Literacy Proficiency |  | 0.397^***^ |  | 0.313^***^ |
|  |  | (0.0417) |  | (0.0570) |
|  |  |  |  |  |
| Life-Wide Reading Engagement |  |  | 0.227^***^ | 0.104^*^ |
|  |  |  | (0.0417) | (0.0456) |
|  |  |  |  |  |
| Age | 0.118^***^ | 0.180^***^ | 0.115^***^ | 0.0998^*^ |
|  | (0.0336) | (0.0352) | (0.0338) | (0.0442) |
|  |  |  |  |  |
| Age-squared | -0.0791^*^ | -0.0533 | -0.0741^*^ | -0.0732 |
|  | (0.0340) | (0.0344) | (0.0341) | (0.0464) |
|  |  |  |  |  |
| Female | 0.0733 | 0.0812 | 0.0901 | 0.210^*^ |
|  | (0.0751) | (0.0754) | (0.0752) | (0.0874) |
|  |  |  |  |  |
| Education | 0.356^***^ | 0.195^***^ | 0.285^***^ | 0.181^***^ |
|  | (0.0323) | (0.0377) | (0.0351) | (0.0469) |
|  |  |  |  |  |
| Native English Speaker | 0.181 | 0.0965 | 0.160 | -0.0625 |
|  | (0.143) | (0.145) | (0.145) | (0.162) |
|  |  |  |  |  |
| NZ Born | -0.0822 | -0.116 | -0.0784 | 0.159 |
|  | (0.0918) | (0.0922) | (0.0929) | (0.0997) |
|  |  |  |  |  |
| Employed | 0.00104 | -0.0741 | -0.104 | 0.277^**^ |
|  | (0.0854) | (0.0865) | (0.0893) | (0.0969) |
|  |  |  |  |  |
| Maori | -0.0499 | -0.0127 | -0.0457 | -0.142 |
|  | (0.0981) | (0.105) | (0.0972) | (0.145) |
|  |  |  |  |  |
| Pasifika | 0.234 | 0.381^*^ | 0.218 | -0.159 |
|  | (0.156) | (0.163) | (0.157) | (0.232) |
|  |  |  |  |  |
| NZ European | -0.0138 | -0.157 | -0.00773 | 0.303 |
|  | (0.132) | (0.131) | (0.133) | (0.168) |
|  |  |  |  |  |
| Asian | -0.199 | -0.110 | -0.201 | -0.0554 |
|  | (0.195) | (0.190) | (0.201) | (0.188) |
|  |  |  |  |  |
| Constant | -0.224 | -0.0234 | -0.145 | -1.660^***^ |
|  | (0.166) | (0.167) | (0.173) | (0.176) |
|  |  |  |  |  |
| N | 4768 | 4768 | 4768 | 4768 |

Standard errors in parentheses

Individuals age 25-65

Literacy Proficiency, Life-Wide Reading Engagement, Age, Education standardised

^*^ *p* < 0.05, ^**^ *p* < 0.01, ^***^ *p* < 0.001
