# Supplementary material for: Adults’ reading engagement and wellbeing in Aotearoa New Zealand
Source: PLoS One. 2023 Sep 28;18(9):e0286706. doi: 10.1371/journal.pone.0286706 (PMC10538774; doi:10.1371/journal.pone.0286706)
Supplement: S6 Table — (DOCX) [file pone.0286706.s006.docx]

**S6 Table. Logit models of high civic engagement.**

|  | Baseline | Literacy | Reading | Full |
| --- | --- | --- | --- | --- |
| Literacy Proficiency |  | 0.190^**^ |  | 0.169^*^ |
|  |  | (0.0664) |  | (0.0670) |
|  |  |  |  |  |
| Life-Wide Reading Engagement |  |  | 0.172^***^ | 0.154^**^ |
|  |  |  | (0.0493) | (0.0499) |
|  |  |  |  |  |
| Age | 0.298^***^ | 0.328^***^ | 0.297^***^ | 0.323^***^ |
|  | (0.0497) | (0.0497) | (0.0505) | (0.0503) |
|  |  |  |  |  |
| Age-squared | -0.0836 | -0.0699 | -0.0794 | -0.0677 |
|  | (0.0447) | (0.0439) | (0.0447) | (0.0438) |
|  |  |  |  |  |
| Female | 0.298^***^ | 0.302^***^ | 0.310^***^ | 0.312^***^ |
|  | (0.0766) | (0.0765) | (0.0784) | (0.0783) |
|  |  |  |  |  |
| Education | 0.299^***^ | 0.222^***^ | 0.244^***^ | 0.181^**^ |
|  | (0.0516) | (0.0576) | (0.0517) | (0.0576) |
|  |  |  |  |  |
| Native English Speaker | -0.0534 | -0.0994 | -0.0741 | -0.112 |
|  | (0.153) | (0.156) | (0.151) | (0.153) |
|  |  |  |  |  |
| NZ Born | -0.0494 | -0.0688 | -0.0530 | -0.0695 |
|  | (0.132) | (0.131) | (0.132) | (0.131) |
|  |  |  |  |  |
| Employed | -0.260^*^ | -0.296^**^ | -0.337^**^ | -0.359^**^ |
|  | (0.106) | (0.110) | (0.109) | (0.113) |
|  |  |  |  |  |
| Maori | 0.221 | 0.244 | 0.233 | 0.253 |
|  | (0.164) | (0.166) | (0.162) | (0.164) |
|  |  |  |  |  |
| Pasifika | 0.418 | 0.490^*^ | 0.404 | 0.470^*^ |
|  | (0.233) | (0.233) | (0.228) | (0.229) |
|  |  |  |  |  |
| NZ European | -0.489^**^ | -0.553^**^ | -0.480^*^ | -0.538^**^ |
|  | (0.188) | (0.187) | (0.187) | (0.186) |
|  |  |  |  |  |
| Asian | -0.697^***^ | -0.655^***^ | -0.699^***^ | -0.661^***^ |
|  | (0.197) | (0.197) | (0.195) | (0.194) |
|  |  |  |  |  |
| Constant | -1.038^***^ | -0.944^***^ | -0.981^***^ | -0.907^***^ |
|  | (0.236) | (0.237) | (0.232) | (0.234) |
|  |  |  |  |  |
| N | 4768 | 4768 | 4768 | 4768 |

Standard errors in parentheses

Individuals age 25-65

Literacy Proficiency, Life-Wide Reading Engagement, Age, Education standardised

^*^ *p* < 0.05, ^**^ *p* < 0.01, ^***^ *p* < 0.001
